# Supplementary figures and images for: PPARα Induces the Expression of CAR That Works as a Negative Regulator of PPARα Functions in Mouse Livers
Source: Int J Mol Sci. 2023 Feb 16;24(4):3953. doi: 10.3390/ijms24043953 (PMC9960678; doi:10.3390/ijms24043953)

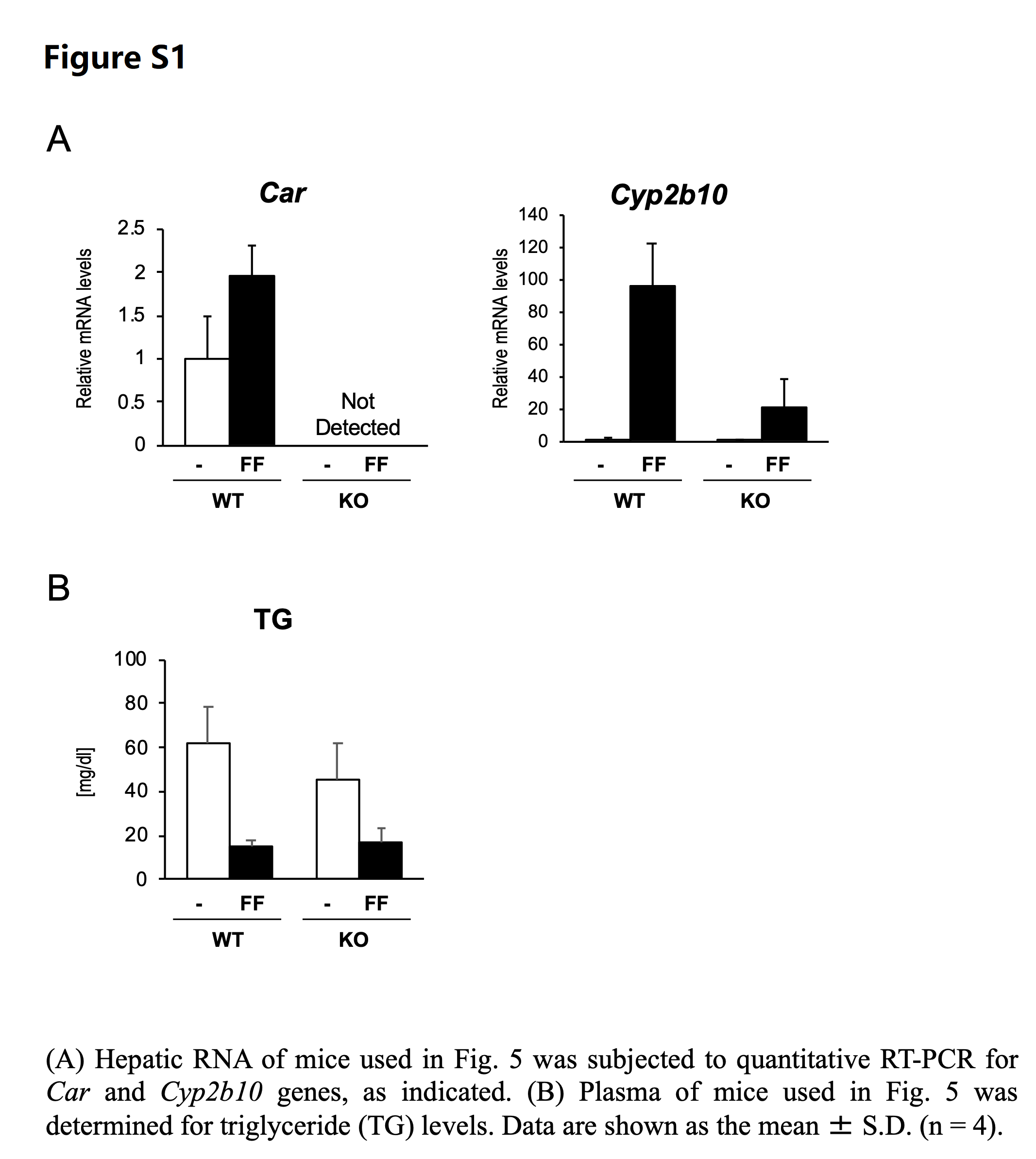

Supplement: Supplementary file 1 [file ijms-24-03953-s001.zip › ijms-2105935-supplementary.tiff]
